# Impact of low skeletal muscle mass and quality on clinical outcomes in patients with head and neck cancer undergoing (chemo)radiation

**DOI:** 10.3389/fnut.2022.994499

**Published:** 2022-11-17

**Authors:** Lilia Bardoscia, Giulia Besutti, Massimo Pellegrini, Maria Pagano, Candida Bonelli, Efrem Bonelli, Luca Braglia, Salvatore Cozzi, Massimo Roncali, Cinzia Iotti, Carmine Pinto, Pierpaolo Pattacini, Patrizia Ciammella

**Affiliations:** ^1^Radiation Oncology Unit, Azienda Unità Sanitaria Locale-IRCCS di Reggio Emilia, Reggio Emilia, Italy; ^2^Radiology Unit, Department of Imaging and Laboratory Medicine, Azienda Unità Sanitaria Locale-IRCCS di Reggio Emilia, Reggio Emilia, Italy; ^3^Department of Medical and Surgical Sciences, University of Modena and Reggio Emilia, Modena, Italy; ^4^Department of Biomedical, Metabolic, and Neural Sciences, University of Modena and Reggio Emilia, Modena, Italy; ^5^Oncology Unit, Azienda Unità Sanitaria Locale-IRCCS di Reggio Emilia, Reggio Emilia, Italy; ^6^Research and Statistics Infrastructure, Azienda Unità Sanitaria Locale-IRCCS di Reggio Emilia, Reggio Emilia, Italy; ^7^Nuclear Medicine Unit, Azienda Unità Sanitaria Locale-IRCCS di Reggio Emilia, Reggio Emilia, Italy

**Keywords:** head and neck cancer, sarcopenia, myosteatosis, muscle quality, muscle quantity, radiotherapy, clinical outcomes, overall survival

## Abstract

The study aimed to explore the impact of low skeletal muscle mass and quality on survival outcomes and treatment tolerance in patients undergoing radical chemo-radiation therapy for head and neck cancer (HNC). This is significant given the growing interest in sarcopenia as a possible negative predictive/prognostic factor of disease progression and survival. From 2010 to 2017, 225 patients were included in the study. Pre-treatment computed tomography (CT) scans of HNC patients undergoing (chemo)radiation therapy were retrospectively reviewed. The skeletal muscle area, normalized for height to obtain the skeletal muscle index (SMI), the skeletal muscle density (SMD) and the intramuscular adipose tissue area (IMAT) were measured at the level of the L3 vertebra. Low SMD and low SMI were defined according to previously reported thresholds, while high IMAT was defined using population-specific cut-point analysis. SMI, SMD, and IMAT were also measured at the proximal thigh (PT) level and tested as continuous variables. Clinical morpho-functional parameters, baseline nutritional markers with a known or suspected impact on HNC treatment, clinical outcomes and sarcopenia were also collected. In multivariate analyses, adjusted by age, sex, stage, diabetes, body mass index (BMI), and weight loss, L3-SMI was not significantly associated with survival, while poor muscle quality was negatively associated with overall survival (OS) (HR = 1.88, 95% CI = 1.09–3.23, *p* = 0.022 and HR = 2.04, 95% CI = 1.27–3.27, *p* = 0.003, for low L3-SMD and high L3-IMAT, respectively), progression-free survival (PFS) (HR = 2.26, 95% CI = 1.39–3.66, *p* = 0.001 and HR = 1.97, 95% CI = 1.30–2.97, *p* = 0.001, for low L3-SMD and high L3-IMAT, respectively) and cancer-specific survival (CSS) (HR = 2.40, 95% CI = 1.28–4.51, *p* = 0.006 and HR = 1.81, 95% CI = 1.04–3.13, *p* = 0.034, for low L3-SMD and high L3-IMAT, respectively). Indices at the PT level, tested as continuous variables, showed that increasing PT-SMI and PT-SMD were significant protective factors for all survival outcomes (for OS: HR for one cm^2^/m^2^ increase in PT-SMI 0.96; 95% CI = 0.94–0.98; *p* = 0.001 and HR for one HU increase in PT-SMD 0.90; 95% CI = 0.85–0.94; *p* < 0.001, respectively). PT-IMAT was a significant risk factor only in the case of CSS (HR for one cm^2^ increase 1.02; 95% CI = 1.00–1.03; *p* = 0.046). In conclusion, pre-treatment low muscle quality is a strong prognostic indicator of death risk in patients affected by HNC and undergoing (chemo)radiotherapy with curative intent.

## Introduction

Head and neck cancers (HNCs) include malignant tumors of the lip, oral cavity, oropharynx, hypopharynx, larynx, nasopharynx, and salivary glands and are responsible for more than 450,000 deaths annually (1). Besides classical risk factors like older age, tumor stage, dietary factors, alcohol and tobacco consumption, as well as HPV status, the assessment of multiple body composition parameters has been recently regarded as an important predictor of clinical outcome (2, 3).

Sarcopenia has been defined as a generalized skeletal muscle disorder associated with an increased likelihood of adverse outcomes (4) and its importance for survival analysis and radio-chemotherapy toxicity in cancer patients has been recognized for different tumors including HNC (3, 5, 6). The diagnosis of sarcopenia is confirmed by the occurrence of a low muscle mass or a low muscle quality, associated with reduced muscle strength and performance (4). The assessment of the CT cross-sectional skeletal muscle area (SMA) or SMA normalized for height to obtain the SMI, at the level of the third lumbar vertebra (L3), is the current gold standard for inferring total skeletal muscle mass (7, 8).

Following CT segmentation at L3, SMD and intermuscular adipose tissue (IMAT) infiltration can be measured, giving an indication of skeletal muscle mass quality. SMD is measured in Hounsfield units (HU), and a lower density highlights that more intramuscular lipid infiltration or myosteatosis is present (8). Increasing IMAT, instead, indicates a higher fat infiltration within the muscle fibers and underneath the fascia, providing another index of poor muscle quality (9).

Besides L3, CT muscle indices in different muscle groups have been used in the assessment of sarcopenia (8, 10–12). We have recently found a good correlation between muscle indices at the PT with OS and PFS in patients with hematologic malignancies (13). Others have studied the predictive power of CT-cross-sectional measurements at the level of the third cervical vertebra in HNC patients (2, 14).

Radiotherapy (RT) plays a key role in the curative treatments of HNC (15). Ganju et al. showed that low muscle mass reduces chemo-radiation therapy (CRT) compliance and increases chemotherapy (CHT) toxicity in patients with locally advanced HNC (16). Grossberg et al. showed that pre- and post-treatment low muscle mass is associated with poorer OS in a cohort of 190 HNC patients, treated with CRT (17): a significant reduction in OS, from 75 to 62%, was observed in sarcopenic patients, by comparison with non-sarcopenic patients. Post-treatment reduction in muscle mass was also associated with a reduction in OS, relative to non-sarcopenic patients (17, 18). Importantly, generalized weight loss was not associated with any significant changes in patient outcomes (6, 17, 18), thus emphasizing the importance of measuring body composition, rather than simply total body weight.

Head and neck cancer (HNC) patients undergo CT evaluations at the baseline and at multiple timepoints during their treatment, thus body composition data may represent a powerful and easily available additional prognostic factor. Should the prognostic value of CT parameters be confirmed, they may also be used to guide interventions based on nutritional support and exercise (19). However, the available experiences of the impact of low muscle quality on cancer patients, in particular HNC patients treated with RT, with or without additional systemic treatment, are still deficient in the literature.

In this retrospective study of the body composition of HNC patients undergoing definitive CRT, we aimed to explore the impact of pre-treatment low muscle mass and quality on survival outcomes and treatment tolerance.

## Materials and methods

### Study design

We conducted a monocentric retrospective study with the primary aim of evaluating the association between baseline skeletal muscle quality and quantity, and OS. Secondary endpoints were: acute toxicity (within 6 months after the end of the treatment) ≥ G3 according to CT-CAE v4.0 Classification (20); treatment compliance with temporary or definitive treatment suspension; local control of the disease, defined as the absence of any clinical or radiological evidence of local recurrence after complete response to primary treatment until the last follow up visit; PFS and CSS.

The present study was given final approval by the Area Vasta Emilia Nord Ethical Committee (AUSLRE protocol number 2019/0066663 on 05/06/2019 and AUSLRE protocol number 2021/0070072 on 28/05/2021) and was performed in accordance with the principles of Good Clinical Practice (GCP) in respect of the ICH GCP guidelines and the ethical principles contained in the Helsinki Declaration and its subsequent updates (21). Given the retrospective nature of the data collection, the Ethics Committee authorized the use of a patient’s data without his/her informed consent if all reasonable efforts have been made to contact that patient to obtain it.

### Patient selection and treatment

All consecutive patients with histologically confirmed HNC, undergoing definitive RT with or without concurrent CHT or induction CHT and subsequent chemo-radiation, who were treated with curative intent at our institution between January 2010 and December 2017, were eligible. The diagnostic work up for all the recruited patients included total body 20-deoxy-20-[18F] fluoro-*D*-glucose (FDG) positron emission tomography, in combination with a CT scan (PET-CT), a contrast-enhanced CT (CE-CT) of the head, neck and chest, with or without magnetic resonance (MRI).

Data regarding patient age, gender, cancer type, tumor stage, tumor site, Eastern Cooperative Oncology Group (ECOG) score, smoking status, alcohol use, total protein level, albumin level, glycemia, lactate dehydrogenase (LDH), human papillomavirus (HPV) p16 status, tumour-node-metastasis (TNM) stage (22), Charlson Comorbidity Index (CCI) (23), therapeutic details and any complications experienced during and after treatment were obtained from the patients’ medical records.

Following CRT, patients started a regular follow up (FU) at our institution: every 2 months for the first year, then every 3 to 4 months during the second year; every 4 to 6 months 3 years after the treatment and every 6 months to the 1-year FU (for early-stage disease only) until the fifth year after the treatment. Each FU visit included an interview for the assessment of CTCAE v4.02 ear-nose-throat (ENT)-related symptoms (20), ENT clinical examination and flexible endoscopy. A restaging of the disease with 18F-FDG PET-CT was required 3 months after the end of (chemo)radiation, then a neck and chest CT scan was carried out annually and, in some cases, a head and neck MRI was performed every four to 6 months until the end of the FU period.

### Computed tomography muscle assessment

Skeletal muscle quality and quantity were evaluated on staging, pre-treatment CT scan imaging, using images acquired without contrast media administration during the PET-CT scan. Manual segmentation of the skeletal muscle at the level of the L3 was performed by a single trained operator under the supervision of a senior radiologist, using the commercially available software package, Osirix, after having applied a radiodensity range between -29 and +150 HUs, which is specific for muscle tissue. The lean muscle cross-sectional area was normalized for the squared height to obtain the SMI. SMD was collected for the same region of interest selected for lean muscle cross-sectional area measurement. The IMAT area (the fat area between muscle fibers and within the fascia) was measured by applying a density range between −180 and −30 HU, thresholds specific for fat tissue. Low SMD and low SMI were defined according to previously reported threshold values (SMD < 41 HU for BMI < 25, < 33 HU for BMI ≥ 25; SMI < 41 cm^2^/m^2^ in women and < 43 cm^2^/m^2^ or < 53 cm^2^/m^2^ in men, with a BMI < 25 or ≥ 25 respectively), while high IMAT was defined using population-specific, cut-point analysis (Supplementary Table 1) (11). SMI, SMD and IMAT were also measured at the PT level, as previously reported, when included in the PET-CT scan (13).

### Statistical analysis

In the absence of *a priori* hypothesis and given the exploratory nature of the study, no formal sample size calculation was performed. Clinical and demographic data were expressed in terms of frequency and percentage for categorical variables, median and interquartile range (IQR) for quantitative variables. The project’s main aim was to test the prognostic value of Martin et al.’s cut-offs for SMI and SMD on treatment interruption/response and survival outcomes; furthermore, we researched cut-offs for IMAT in an exploratory way in our sample. OS time was measured from the time that RT ended until death or the last FU. We also estimated cancer specific survival (CSS), which differs from OS in terms of non-cancer-death censoring. Finally, PFS was calculated from RT ending to relapse or death, whichever came first, or to the last FU. Optimal cut-point analysis for IMAT, targeted to OS and split by BMI and age, followed the methodology by Contal and O’Quigley (24). The association between markers and CHT/RT interruption, severe AE (CTCAE ≥ 3) and local control was estimated with logistic regressions. Survival functions were estimated using the Kaplan-Meier method. The association between markers and survival outcome was estimated with univariate and multivariate Cox regressions. Proportional hazard assumption was assessed by testing scaled Schoenfeld residuals’ correlation with time; no violation of the assumption was found. Unless otherwise specified, confidence intervals (CIs) were two-tailed and calculated considering a 0.95 confidence level. Performed tests were considered statistically significant if the *p*-values were < 0:05. Statistical analysis was performed using R 3.5.2 R Core Team (2021).

## Results

### Clinical characteristics of the patients

A total of 225 consecutive patients diagnosed with histologically confirmed HNC undergoing treatment between January 2010 and December 2017 were included in the present study. Patients, tumor and treatment characteristics are summarized in Table 1. Among the 225 included patients, 170 (75.6%) were male; the median age was 64.5 years (IQR 56.3–72.4 years); the median baseline BMI was 24.8, with substantial weight stability in the 6 months before CRT (weight loss: median 0, third quartile 1 kg). According to some of the parameters that define malnutrition (25), we found that 42 (18.67%) patients lost > 5% body weight in the 6 months before diagnosis and 31 (13.78%) patients had a low BMI in relation to age (BMI < 20 in patients < 70 years old and BMI < 22 in patients ≥ 70 years old), for a total of 60 (26.7%) patients with at least one of the two parameters. Nearly 70% of patients were smokers. Of the 225 patients, 98 (43.8%) showed good performance status with the ECOG 0 or 1, while 97 (43.3%) showed intermediate performance status (ECOG > 1). Blood test results are reported in Supplementary Table 2.

**TABLE 1:** Study cohort description, including patients’ clinical characteristics, main cancer features, radiation treatment type, and main outcomes.

		Patients (*n* = 225)
Sex	Female, n (%)	55 (24.4)
	Male, n (%)	170 (75.6)
Age (years) median (IQR)		64.5 (56.3–72.35)
	≥ 60 years old, n (%)	146 (65)
	< 60 years old, n (%)	79 (35)
BMI, median (IQR)		24.6 (22.15–27.4)
Weight loss in the previous 6 months (kg) median (IQR)		0 (0–1)
Comorbidities, n (%)	Hypertension	85 (37.8)
	DM	28 (12.4)
	COPD	18 (8)
	Alcohol abuse	14 (6.2)
PS ECOG, n (%)	0	98 (43.75)
	1	97 (43.3)
	2	26 (11.6)
	3	3 (1.34)
Smoke, n (%)		156 (69.3)
Tumor site, n (%)	Oral cavity	12 (5.3)
	Hypopharynx	35 (15.6)
	Larynx	38 (16.9)
	Oropharynx	101 (44.9)
	Occult primary	10 (4.4)
	Nasopharynx	28 (12.4)
	Paranasal Sinuses	1 (0.4)
TNM stage, n (%)	I	11 (4.9)
	II	23 (10.2)
	III	52 (23.2)
	IV	1 (0.4)
	IVa	127 (56.49)
	IVb	11 (4.9)
HPV status, n (%)	Positive	49 (22.0)
	Negative	48 (21.5)
	Unknown	126 (56.5)
EBV status, n (%)	Positive	8 (3.6)
	Negative	3 (1.4)
	Unknown	212 (95)
	Chemoradiation (CRT)	124 (55.1)
Treatment type, n (%)	CRT after induction CHT	42 (18.7)
	Radiotherapy alone	57 (25.3)
Concomitant CHT regimen	Platinum-based (CDDP)	136 (80.9)
	Cetuximab	30 (17.9)
	1	19 (8.4)
Acute toxicity	2	151 (67.1)
CTCAE v4.0 grade, n (%)	3	54 (24)
	4	1 (0.4)
RT suspension > 10 days, n (%)		134 (79.8)
	Complete Response	176 (78.2)
	Partial Response	10 (4.4)
Response to therapy, n (%)	Stable Disease	23 (10.2)
	Progressive Disease	9 (4)
	Not evaluable	7 (3.1)
Local recurrence, n (%)		62 (27.6)
Death, n (%)		96 (42.7)
	HNC	71 (74)
Cause of death, n (% on	Toxicity	2 (2)
total deaths)	Other	23 (24)

Continuous variables are presented as median and interquartile range while categorical data are reported as frequency and percentage. BMI, body mass index; DM, diabetes mellitus; COPD, chronic obstructive pulmonary disease; CHT, chemotherapy; CTCAE, Common Terminology Criteria for Adverse Events; RT, radiotherapy; HNC, head and neck cancer.

The tumor site was most frequently oropharynx (44.9%), followed by larynx (16.9%), hypopharynx (15.5%), and nasopharynx (12.4%), and the most represented stage was III/IV (84.9% of cases) according to TNM. Histologically, 193 tumors (85.8%) were squamous cell carcinoma (SCC), while five (2.2%) patients exhibited a histology other than SCC (i.e., lymphoepithelioma-like carcinoma of the nasopharynx, non-keratinizing carcinoma, adenocarcinoma). Positive HPV staining was found in 49 (22%) of the patients.

Regarding treatment options, RT was mainly administered using intensity-modulated techniques, such as volumetric multiple arc therapy (VMAT) in 35 (15.5%) patients and helical tomotherapy was used in 176 (78.2%) patients with an average number of 31 sessions; of these 176 patients, 124 (55.1%) received this treatment concurrently with CHT. Altered fractionation 2.12 Gy up to 2.35 Gy/fraction was preferred (EQD2Gy 66 to 70 Gy). Platinum-based regimens were more frequently used as concomitant CHT and the anti-EGFR drug, cetuximab, was offered as an alternative option in a minority of patients (17.9%). Forty-four (19.6%) patients underwent induction CHT and a combination of fluoropyrimidine and cisplatin with and without taxanes was administered to six (13.6%) and 38 (86.4%) patients, respectively. RT was suspended for more than 10 days in 13 (5.8%) patients, due to the worsening of their general health, while concurrent CHT was suspended due to complications in 34 (20.2%) patients.

Grade 3 and 4 acute toxicity was reported in 55 (24.4%) patients. After a median FU time of 5.6 years (95% CI 5.0–6.4), 96 (42.7%) patients had died, 71 (74%) of them due to HNC-related causes. After therapy, 176 (78.22%) patients were in complete remission and 62 patients exhibited a recurrence of the disease during their FU.

### Association between muscle quality and quantity and patient outcomes

**FIGURE 1:**
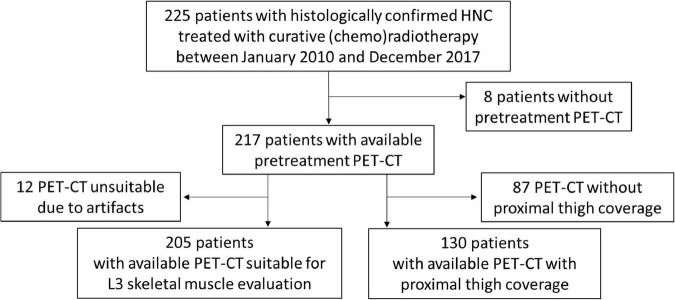
Patient inclusion flow-chart. Flow-chart representing patient inclusion and subgroups of patients with available L3-and proximal thigh (PT)-computed tomography (CT) parameters of muscle quantity and quality.

Among the 225 patients included in the study, the baseline PET-CT scan was not available in the case of eight patients and was not suitable for muscle quality and quantity assessment at the L3 level in 12 patients due to artifacts, while the CT images at the PT level were only available for 130 patients (Figure 1). The distribution of muscle quality and quantity parameters, assessed by the CT scans, is reported in Table 2, as well as the prevalence estimates of sarcopenia applying cutoffs provided by Martin et al. (11) and by our cut-point analysis on IMAT (see Supplementary Table 1).

**TABLE 2:** Distribution of computed tomography (CT) parameters of skeletal muscle quality and quantity, and prevalence of sarcopenia according to different parameters and different cut-off values.

CT body composition parameters and prevalence of sarcopenia		
L3-SMD (HU), median (IQR) (*n* = 203)		39.5 (34.0–44.0)
L3-SMI (cm^2^/m^2^), median (IQR) (*n* = 205)		52.11 (46.0–59.1)
L3-IMAT (cm^2^), median (IQR) (*n* = 203)		10.5 (7.0–17.8)
PT-SMD (HU), median (IQR) (*n* = 130)		50.0 (47.0–53.0)
PT-SMI (cm^2^/m^2^), median (IQR) (*n* = 130)		83.8 (72.3–92.9)
PT-IMAT (cm^2^), median (IQR) (*n* = 130)		18.0 (10.0–28.0)
Prevalence of sarcopenia, n (%), (95% CI)	L3-SMD Martin’s cut-offs^a^	84 (40.0%), (33.32–47.0%)
	L3-SMI Martin’s cut-offs^a^	49 (23.3%), (17.8–29.7%)
	L3-IMAT cut-point analysis cut-offs^b^	97 (47.1%), (40.1–54.2%)

L3, third lumbar vertebrae; SMD, skeletal muscle density; SMI, skeletal muscle index; IMAT, intermuscular adipose tissue; PT, proximal thigh; IQR, interquartile range; CI, confidence interval.

^a^Cut-offs according to Martin et al. (11).

^b^Cut-offs according to cut-point analysis on our population.

#### Short-term outcomes

Univariate logistic models for short-term outcomes including RT and CHT suspension, CTCAE ≥ 3 events and local control, showed no statistically significant association between low muscle quality and quantity and outcomes (Table 3). However, from a clinical standpoint, low muscle quality, defined as lower-than-threshold SMD or higher-than-threshold IMAT was positively associated with treatment suspension and CTCAE ≥ 3 events. For example, the ORs of high IMAT (compared to low IMAT) were 1.78 (95% CI = 0.7–4.7, *p* = 0.23) for RT suspension, and 1.96 (95% CI 0.87–4.49, *p* = 0.11) for CHT suspension, while the OR of low SMD (compared to high SMD) for CTCAE ≥ 3 events was 1.40 (95% CI = 0.74–2.64, *p* = 0.30). Lower-than-threshold L3-SMD also showed a not statistically significant association with diminished local disease control (OR = 0.56; 95% CI = 0.29–1.09; *p* = 0.09).

**TABLE 3:** Univariate logistic regressions between low muscle quantity and low muscle quality with short-term outcomes.

Short-term outcomes				

		OR	95% CI	*P*-value
RT suspension	Low L3-SMD (Martin)^a^	1.28	0.52–3.13	0.58
	Low L3-SMI (Martin)^a^	0.96	0.30–2.59	0.94
	High L3-IMAT (cut-point)^b^	1.78	0.70–4.74	0.23
CHT suspension	Low L3-SMD (Martin)^a^	1.17	0.52–2.61	0.70
	Low L3-SMI (Martin)^a^	0.83	0.29–2.10	0.70
	High L3-IMAT (cut-point)^b^	1.96	0.87–4.49	0.11
CTCAE ≥ 3	Low L3-SMD (Martin)^a^	1.40	0.74–2.64	0.30
	Low L3-SMI (Martin)^a^	0.98	0.45–2.02	0.96
	High L3-IMAT (cut-point)^b^	1.30	0.69–2.47	0.42
Local disease control	Low L3-SMD (Martin)^a^	0.56	0.29–1.09	0.09
	Low L3-SMI (Martin)^a^	1.28	0.59–3.03	0.55
	High L3-IMAT (cut-point)^b^	0.81	0.42–1.58	0.54

Univariate logistic regressions between low muscle quantity (low L3-SMI) and low muscle quantity (low L3-SMD or high L3-IMAT) and short-term outcomes including RT and CHT suspension, toxicity (CTCAE ≥ 3 events), and complete response to therapy. OR, odds ratio; CI, confidence interval; L3, third lumbar vertebrae; SMD, skeletal muscle density; SMI, skeletal muscle index; IMAT, intermuscular adipose tissue.

^a^Cut-offs according to Martin et al. (11).

^b^Cut-offs according to cut-point analysis on our population.

#### Survival

**FIGURE 2:**
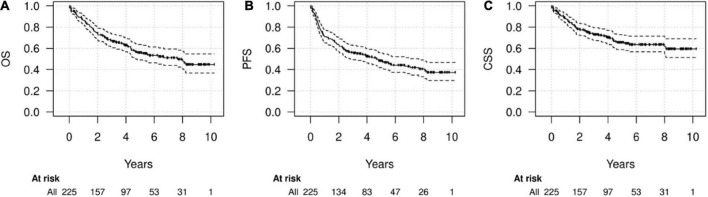
Survival of the whole cohort. Kaplan-Meier diagrams for overall survival (OS) **(A)**, progression-free survival (PFS) **(B)**, and cancer-specific survival (CSS) **(C)** in the whole cohort.

During FU (median 5.6 years), 62 recurrences and 96 deaths (71/96 caused by HNC) were recorded. The median OS was 7.6 years (95% CI 4.7-NA) while median PFS was 4.7 years (95% CI 2.8–7.6). Five-year and 10-year OS were 55.9% (49.1–63.6%) and 44.8% (36.7–54.7%), respectively; five-year and 10-year PFS were 48.3% (41.8–55.9) and 37.3% (29.8–46.7%), respectively and 5-year and 10-year CSS were 65.6% (58.9–73%) and 59.5% (51.3–69%), respectively (Figure 2).

**FIGURE 3:**
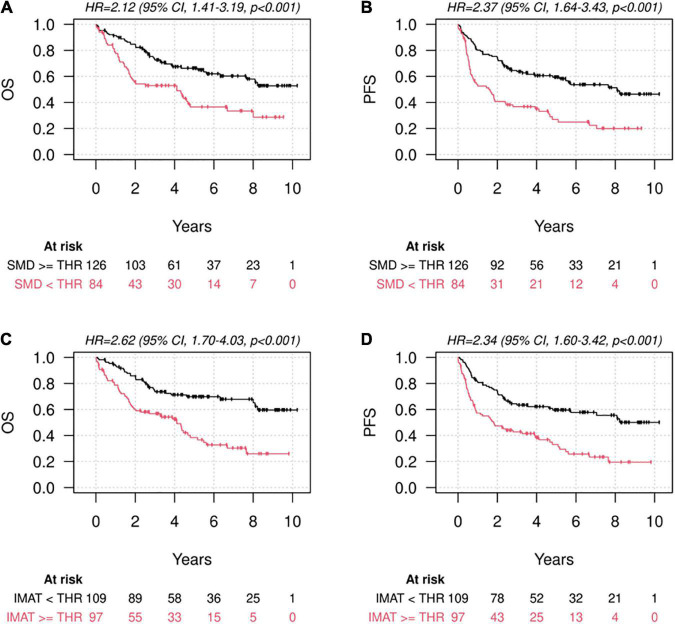
Survival according to muscle quantity and quality. Kaplan-Meier diagrams for Overall Survival (OS) **(A,C)** and Progression-Free Survival (PFS) **(B,D)**, subdivided by higher-and lower-than-thresholds skeletal muscle density at the level of L3 (L3-SMD) according to the cut-offs defined by Martin et al. (11) **(A,B)** and higher-and lower-than-thresholds intermuscular adipose tissue area at the level of L3 (L3-IMAT), according to the cut-offs defined by means of cut-point analysis on our population **(C,D)**.

Univariate analyses showed a significant association of low muscle quality (in terms of both lower-than-thresholds L3-SMD and higher-than-thresholds L3-IMAT) with lower survival rates, while lower-than-thresholds L3-SMI (low muscle quantity) was not significantly associated with survival (Figure 3 and Table 4). These results were confirmed in multivariate analyses adjusted for other prognostic factors, including age, sex, stage, diabetes, BMI, and weight loss in the previous 6 months. In particular, no significant association was found for low muscle quantity, while both low L3-SMD and high L3-IMAT were associated with OS (HR = 1.88, 95% CI = 1.09–3.23, *p* = 0.022 and HR = 2.04, 95% CI = 1.27–3.27, *p* = 0.003, for low L3-SMD and high L3-IMAT, respectively), PFS (HR = 2.26, 95% CI = 1.39–3.66, *p* = 0.001, and HR = 1.97, 95% CI = 1.30–2.97, *p* = 0.001, for low L3-SMD and high L3-IMAT, respectively) and CSS (HR = 2.40, 95% CI = 1.28–4.51, *p* = 0.006 and HR = 1.81, 95% CI = 1.04–3.13, *p* = 0.034, for low L3-SMD and high L3-IMAT, respectively). As for other prognostic factors (results not shown), covariate HRs suggested a detrimental prognostic effect for higher age, stage III-IV, male sex, having diabetes, lower BMI and higher weight loss in the previous 6 months; regarding these factors, the associations were not statistically significant for all the estimates but had a common direction.

**TABLE 4:** Univariate and multivariate associations between low muscle quantity (low L3-SMI) and low muscle quality (low L3-SMD or high L3-IMAT) and OS, PFS, or CSS.

	Univariate analyses			Multivariate analyses (Adjusted by age, sex, stage, diabetes, BMI, and previous weight loss)		
		
	HR	95% CI	*P*-value	HR	95% CI	*P*-value
***Overall survival***						
Low L3-SMD (Martin)^a^	2.12	1.41–3.19	< 0.001	1.88	1.09–3.23	0.022
Low L3-SMI (Martin)^a^	1.19	0.74–1.93	0.47	0.86	0.50–1.47	0.574
High L3-IMAT (cut-point)^b^	2.62	1.71–4.03	< 0.001	2.04	1.27–3.27	0.003
***Progression free survival***						
Low L3-SMD (Martin)^a^	2.37	1.64–3.43	< 0.001	2.26	1.39–3.66	0.001
Low L3-SMI (Martin)^a^	1.18	0.78–1.81	0.45	0.84	0.51–1.36	0.469
High L3-IMAT (cut-point)^b^	2.34	1.60–3.42	< 0.001	1.97	1.30–2.97	0.001
***Cancer-specific survival***						
Low L3-SMD (Martin)^a^	2.46	1.53–3.96	< 0.001	2.40	1.28–4.51	0.006
Low L3-SMI (Martin)^a^	1.25	0.72–2.16	0.43	0.94	0.51–1.74	0.854
High L3-IMAT (cut-point)^b^	2.56	1.56–4.23	< 0.001	1.81	1.04–3.13	0.034

Multivariate associations were adjusted by age, sex, HNC stage (III and IV vs. I and II), diabetes, BMI, and previous weight loss. HR, Hazard Ratio; CI, confidence interval; L3, third lumbar vertebrae; SMD, skeletal muscle density; SMI, skeletal muscle index; IMAT, intermuscular adipose tissue; PT, proximal thigh.

^a^Cut-offs according to Martin et al. (11).

^b^Cut-offs according to cut-point analysis on our population.

Since indices assessed at the PT level were not used to define muscle quality and quantity according to specified cut-offs, due to the high proportion of missing values, they were tested as continuous variables in multivariable Cox models for survival, adjusted by age, sex, BMI, and stage (Table 5). At the PT level, increasing muscle quantity defined by PT-SMI was a significant protective factor (HR for one cm^2^/m^2^ increase 0.96; 95% CI = 0.94–0.98; *p* = 0.001 for OS). Increasing muscle quality, described by increasing PT-SMD was also protective (HR for one HU increase 0.90; 95% CI = 0.85–0.94; *p* < 0.001 for OS), while increasing PT-IMAT was a significant risk factor (and at a borderline level) only in the case of CSS (HR for one cm^2^ increase 1.02; 95% CI = 1.00–1.03; *p* = 0.046).

**TABLE 5:** Multivariate associations of indices of low muscle quality and quantity at the PT level (used as continuous variables) with OS, PFS, and CSS.

	HR	95% CI	*P*-value
***Overall survival***			
PT-SMD (for one HU increase)	0.90	0.85–0.94	< 0.001
PT-SMI (for one cm^2^/m^2^ increase)	0.96	0.94–0.98	0.001
PT-IMAT (for one cm^2^ increase)	1.01	1.00–1.03	0.132
***Progression-free survival***			
PT-SMD (for one HU increase)	0.92	0.87–0.96	< 0.001
PT-SMI (for one cm^2^/m^2^ increase)	0.97	0.95–0.99	0.006
PT-IMAT (for one cm^2^ increase)	1.00	0.99–1.02	0.80
***Cancer-specific survival***			
PT-SMD (for one HU increase)	0.89	0.84–0.94	< 0.001
PT-SMI (for one cm^2^/m^2^ increase)	0.96	0.93–0.99	0.007
PT-IMAT (for one cm^2^ increase)	1.02	1.00–1.03	0.046

Adjusting factors were: age, sex, BMI, and stage. HR, Hazard Ratio; CI, confidence interval; PT, proximal thigh; SMD, skeletal muscle density; SMI, skeletal muscle index; IMAT, intermuscular adipose tissue area.

Similar models on the same subgroup of patients and with parameters of muscle quality/quantity used as continuous variables, were also evaluated for L3-SMD, L3-SMI, and L3-IMAT (Supplementary Table 3). In this subgroup analysis, increasing L3-SMI did not exhibit any protective effect, as opposed to PT-SMI.

## Discussion

The key finding in our study is that pre-treatment low muscle quality is a convincing prognostic indicator of a death risk in patients affected by HNC, undergoing RT or chemo-RT with curative intent. In fact, this retrospective study on 225 patients showed that a high intramuscular fat depot and high IMAT accumulation represent important risk factors for OS and CSS, as well as for disease progression in HNC patients. Indeed, low SMD and high IMAT at L3 were significantly associated with OS, PFS and CSS, and these data were confirmed in multivariate analyses adjusted for other prognostic factors including age, sex, stage, diabetes and BMI. In our study, skeletal muscle mass did not represent a prognostic factor and pre−treatment L3-SMI was not associated with clinical outcome. In this regard the predictive role of skeletal muscle quantity in HNC patients is still being debated. In a metanalysis of seven studies, Takenaka et al. found that sarcopenia, defined as low SMI at L3, predicted OS but the timing of sarcopenia assessment was not reported (2). Other metanalyses showed that radiologically defined sarcopenia was a negative predictor of OS (26–28). Findlay and colleagues in a study of 79 HNC patients found that post-treatment but not pre-treatment low SMI predicted reduced OS on multivariate analysis, with no difference in terms of RT or CHT treatment completion (6). The same author in a subsequent study on 277 HNC patients found that the association between low muscle quantity with OS was not significant on adjusted analysis (29). The discrepancies of the results among different studies may be attributed to the heterogeneity amongst the analyses with a lack of consensus regarding sarcopenia assessment, the different SMI threshold values that were applied or the dissimilar ethnicities of the patients.

Skeletal muscle radiation attenuation or density is another index of muscle status and represents a measure of intramuscular lipid depot or myosteatosis. Myosteatosis is an established, poor prognostic factor in many cancers (30), however, there is a scarcity of studies in patients with HNC. Findlay and colleagues (6) found that pre-treatment myosteatosis predicted reduced OS, and Yoshimura et al. similarly described an association between higher intramuscular adipose tissue content and reduced survival (31), however, in another study on 277 patients the same author did not find a significant association between myosteatosis and OS (29).

The present study on 225 HNC patients confirms that a low SMD is significantly associated with reduced survival even after adjusting for other prognostic factors and introduces important elements of novelty.

The first new fact is the CT assessment of IMAT. The clinical significance of IMAT in oncological patients has been reported (13, 32) but to the best of our knowledge, this is the first study that considers IMAT as a radiological marker of muscle quality and a predictor of clinical outcome in HNC patients. Muscle function and strength are essential elements in the clinical diagnosis of sarcopenia. In this regard, a low muscle quality (low IMAT and/or high SMD) might be a better surrogate marker of muscle function than muscle mass itself (9, 33). In our group of patients, a high IMAT was a predictor of lower OS, PFS, and CSS, similar to a low SMD.

Another novel element is the anatomical site of CT body composition assessment. Besides L3, we have found that muscle status at the PT level represents a valuable prognostic indicator in HNC patients. Indeed, CT-muscle indices of a higher muscle quality or quantity at the PT, tested as continuous variables in multivariable analysis for the absence of specific cut-offs in literature, represented a significant protective factor. Accordingly, in a recent paper we have shown a significant association between PT muscle indices and survival in patients affected by hematological malignancies (13). Muscle status at the PT level could better denote the physical performance status of the patient and thus represent a better marker of the severity of the sarcopenic status (34–36). In particular, it is remarkable that the increase in muscle quantity, as defined by PT-SMI, denoted a significant protective factor differently from L3-SMI.

As for shorter term outcomes, lower-than-threshold L3-SMD showed a weak inverse association with local disease control, while treatment suspension and CTCAE ≥ 3 events were only weakly associated with SMD, confirming the data reported by others (6).

Besides these strengths, the limitations of this study include the retrospective design which needs future confirmation in prospective studies and the high proportion of patients with missing CT scans with PT coverage. The parameters of muscle quality/quantity at PT level were used as continuous variables for the absence of established cut-offs values. To overcome possible interpretation biases, similar models with continuous variables were applied on the same subgroup of patients, also including skeletal muscle parameters at L3 level, confirming the results obtained with the cut-offs. In fact, even in this case, L3-SMI did not show any protective effect, as opposed to PT-SMI.

In conclusion, our study emphasizes that CT−muscle status evaluation, which can be obtained using examinations routinely performed in clinical practice, has a meaningful prognostic value for HNC patients from a clinical perspective. We found that low muscle quality rather than muscle mass was associated with decreased survival and disease progression. Future prospective studies will be necessary to confirm the potential clinical utility of CT muscle assessment for the identification of patients in need of nutritional or pharmacological intervention to fight sarcopenia and to improve muscle quality.

## Data availability statement

The data underlying this study are available on request for researchers intending to conduct research and respect confidentiality (even if anonymous data are provided, they should be published in aggregated form) in studies with objectives consistent with those of the original study. In order to obtain the data, approval must be obtained from the Area Vasta Emilia Nord (AVEN) Ethics Committee which will check the consistency of the objective and planned analyses and will then authorize the authors to provide aggregated or anonymized data.

## Ethics statement

The studies involving human participants were reviewed and approved by Area Vasta Emilia Nord Ethical Committee. The patients/participants provided their written informed consent to participate in this study. Given the retrospective nature of the data collection, the Ethics Committee authorized the use of a patient’s data without his/her informed consent if all reasonable efforts have been made to contact that patient to obtain it.

## Author contributions

LBa, GB, MPe, CI, CP, PP, and PC contributed to the study concept and design. MPa, CB, SC, EB, and MR collected the data. LBr conducted the data analysis. LBa, GB, MPe, EB, and PC interpreted the results. All authors critically reviewed and approved the final version of the manuscript to be submitted.
