# Supplementary material for: Impact of low skeletal muscle mass and quality on clinical outcomes in patients with head and neck cancer undergoing (chemo)radiation
Source: Front Nutr. 2022 Nov 17;9:994499. doi: 10.3389/fnut.2022.994499 (PMC9715267; doi:10.3389/fnut.2022.994499)
Supplement: Supplementary file 1 [file Table_1.DOCX]

Impact of low skeletal muscle mass and quality on clinical outcomes in patients with head and neck cancer undergoing (chemo)radiation

Supplementary Material

**Supplementary Table 1**: Cut-offs to define low muscle quantity and low muscle quality based on L3-SMD, L3-SMI and L3-IMAT

|  | Cut-offs^a^ | | Cut-point analysis |
| --- | --- | --- | --- |
|  | L3-SMD | L3-SMI | L3-IMAT |
| Men with BMI <25 | <41 HU | <43 cm^2^/m^2^ | ≥7 cm^2^/m^2^ |
| Men with BMI ≥25 | <33 HU | <53 cm^2^/m^2^ | ≥14 cm^2^/m^2^ |
| Women with BMI <25 | <41 HU | <41 cm^2^/m^2^ | ≥13 cm^2^/m^2^ |
| Women with BMI ≥25 | <33 HU | <41 cm^2^/m^2^ | ≥29 cm^2^/m^2^ |

BMI, body mass index; SMD, skeletal muscle density; SMI, skeletal muscle index; IMAT, intermuscular adipose tissue area. ^a^Cut-offs according to Martin et al. (10); ^b^cut-offs according to cut-point analysis on our population.

**Supplementary Table 2.** Blood test data of the included patients.

|  | **Patients (n=225)** |
| --- | --- |
| Total Protein level (g/dl) mean (SD) | 7.23 (0.53) |
| Albumin level (g/dl) mean (SD) | 4.31 (0.42) |
| Glycemia (mg/dl) mean (SD) | 107.80 (40.08) |
| Lactate dehydrogenase (LDH) (U/l) mean (SD) | 328.48 (79.78) |

SD, Standard Deviation.

**Supplementary Table 3**. Multivariate associations of indices of low muscle quantity and quality at the proximal thigh level and L3 level (used as continuous variables) with overall, progression-free, and cancer-specific survival, in the subgroup of 130 patients with available indices at PT level

|  | **PT level (n=130)** | | | **L3 level (n=130)** | | |
| --- | --- | --- | --- | --- | --- | --- |
|  | **HR** | **95%CI** | **p-value** | **HR** | **95%CI** | **p-value** |
| *Overall Survival* |  |  |  |  |  |  |
| SMD (for one HU increase) | 0.90 | 0.85-0.94 | <0.001 | 0.94 | 0.91-0.97 | <0.001 |
| SMI (for one cm^2^/m^2^ increase) | 0.96 | 0.94-0.98 | 0.001 | 0.99 | 0.94-1.03 | 0.49 |
| IMAT (for one cm^2^ increase) | 1.01 | 1.00-1.03 | 0.132 | 1.04 | 1.01-1.07 | 0.01 |
| *Progression-Free Survival* |  |  |  |  |  |  |
| SMD (for one HU increase) | 0.92 | 0.87-0.96 | <0.001 | 0.96 | 0.93-0.99 | 0.015 |
| SMI (for one cm^2^/m^2^ increase) | 0.97 | 0.95-0.99 | 0.006 | 0.99 | 0.95-1.03 | 0.50 |
| IMAT (for one cm^2^ increase) | 1.00 | 0.99-1.02 | 0.80 | 1.03 | 1.01-1.06 | 0.02 |
| *Cancer-Specific Survival* |  |  |  |  |  |  |
| SMD (for one HU increase) | 0.89 | 0.84-0.94 | <0.001 | 0.93 | 0.89-0.97 | <0.001 |
| SMI (for one cm^2^/m^2^ increase) | 0.96 | 0.93-0.99 | 0.007 | 0.98 | 0.93-1.03 | 0.41 |
| IMAT (for one cm^2^ increase) | 1.02 | 1.00-1.03 | 0.046 | 1.04 | 1.01-1.08 | 0.013 |

Adjusting factors were: age, sex, BMI, and stage. HR, Hazard Ratio; CI, confidence interval; PT, proximal thigh; L3, third lumbar vertebra; SMD, skeletal muscle density; SMI, skeletal muscle index; IMAT, intermuscular adipose tissue area.
